# Foetal bovine serum influence on in vitro extracellular vesicle analyses

**DOI:** 10.1002/jev2.12061

**Published:** 2021-01-25

**Authors:** Brandon M. Lehrich, Yaxuan Liang, Massimo S. Fiandaca

**Affiliations:** ^1^ Medical Scientist Training Program University of Pittsburgh School of Medicine and Carnegie Mellon University Pittsburgh Pennsylvania USA; ^2^ Center for Biological Science and Technology, Advanced Institute of Natural Sciences Beijing Normal University at Zhuhai Zhuhai China; ^3^ Brain Neurotherapy Bio, Inc Oakland California USA

**Keywords:** cell culture media, EV‐depleted fbs, exosomes, extracellular vesicles, foetal bovine serum, in vitro, serum‐free media

## 1 INTRODUCTION

Extracellular vesicles (EVs) are nanosized lipid bilayer vesicles most notably from either endosomal (i.e., exosomes) or plasma membrane origins (i.e., microvesicles/ectosomes) and released from nearly all mammalian cells (Colombo et al., 2014). An interest in EV research has increased over the past decade, in part due to their participation in complex intercellular communication (Roy et al., 2018). Though EVs are abundant in blood and other biofluids, the investigation of in vitro‐derived EVs provides a critical tool for understanding various mechanisms associated with their biogenesis, molecular composition, packaging of specific payloads, and cellular trafficking. Once released, EVs traffic to target cells where they may be taken up to release their payloads via specific mechanisms, and thereby exert their physiological influence (Colombo et al., 2014; Kowal et al., 2014).

Although engineered micelles and liposomes have previously been utilized as lipid nanocarriers (Fiandaca & S., 2013; Fiandaca et al., 2011) for many therapeutic applications, EVs have garnered recent interest as drug delivery vehicles (Elsharkasy et al., 2020). Currently, there exist vastly heterogeneous cell culture conditions for EV production and isolation (Consortium, 2017). Therefore, there is a current need to define more standard cell culture conditions for investigating EVs that may accelerate the translation of therapeutic clinical‐grade EVs (Lener et al., 2015; Lötvall et al., 2014; Théry et al., 2018). Herein, we present a mini‐review on recent investigations reporting the influence of foetal bovine serum (FBS)‐supplemented media formulations on cultured cell physiology, EV production/release, and its contaminating presence of vesicular and non‐vesicular particles. Additionally, we describe potential solutions and provide recommendations to aid in vitro EV investigators.

## 2 CELL CULTURE CONDITIONS FOR EV INVESTIGATIONS: SERUM USAGE AND CONCERNS

An international survey observed 83% of International Society for Extracellular Vesicles (ISEV) respondents utilize conditioned cell culture media as their starting material (Gardiner et al., 2016). FBS is a common additive in cell culture and 52% of ISEV respondents utilize serum‐containing media for downstream EV analyses, with 59% and 57% of those respondents performing in vitro and in vivo functional studies, respectively (Gardiner et al., 2016). Serum usage, in part due to its ill‐defined composition, provides a variety of contaminating particles (e.g., EVs, lipoproteins, and protein complexes, which differ in their physical properties, yet also have similar size, density, and/or RNA components) that confound these investigative results.

## 3 FBS SUPPLEMENTATION AND GENERAL CONCERNS

The growth factors and other constituents within FBS appear to provide a nourishing ecosystem for many cultured cells (Bettger & Mckeehan, 1986). Despite this nourishing milieu, the presence of FBS in culture has raised specific concerns, including the potential introduction of toxins, viral or prion proteins, and mycoplasma, as well as increased culture variability associated with the inconsistency in the FBS manufacturing process (Khodabukus & Baar, 2014; Kirikae et al., 1997; Treadwell, 1963). Moreover, FBS continues to theoretically raise the potential for both xeno‐immunization and inadvertent zoonotic agent transmission when considered in clinical applications (Dessels et al., 2016).

**FIGURE 1:**
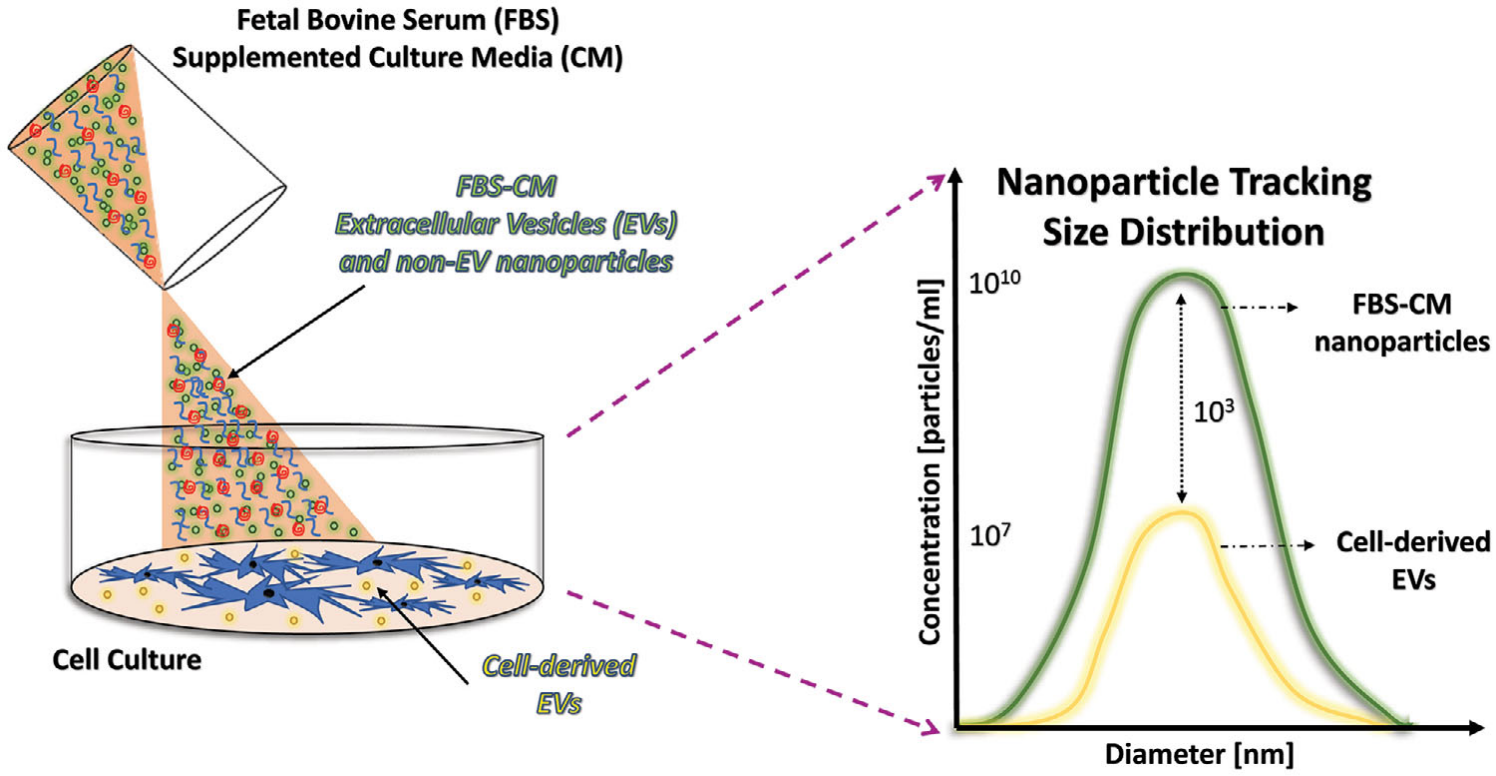
Illustration of FBS‐derived EV and other aggregated non‐EV nanoparticle contamination in conditioned culture media. Specifically, FBS supplies exogenously provided EVs and non‐vesicular contaminants. Adapted from Lehrich et al. Int J Mol Sci, 2018. 19(11)

The major consequence of using native FBS (i.e., untreated FBS that has not undergone any depletion process) to supplement culture media for EV investigations is the requisite introduction of exogenous FBS‐derived EVs and other nanoparticles (e.g., protein/growth factor aggregates) within the population of in vitro‐derived EVs, thereby contaminating the EV fraction available for downstream isolation (Figure 1). Upon EV isolation, the final fraction will contain a mixture of EVs (and potentially other nanoparticles) derived from both the cultured cells and the conditioned media, thereby confounding any in vitro and in vivo analyses. Moreover, cell‐free DNA fragments have been confirmed in FBS and are known to closely associate with FBS‐derived EVs on the surface (Shelke, 2018). Unfortunately, current guidelines for FBS manufacturing do not include the routine testing (or removal) of DNA, rendering its presence uncertain within the cell culture system. The stability of the DNA itself, in combination with the stable conformation between the lipid‐membrane and DNA fragments may further assist cellular uptake of exogenous DNA, subsequently potentially modulating cultured cell physiology (Langecker et al., 2014). In addition, DNA may be found enclosed within the vesicular lumen of FBS‐derived EVs, leaving the possibility for co‐isolation with cell‐derived EVs (Malkin & Bratman, 2020). Moreover, the characteristics of cell‐derived EVs may be affected by the presence of exogenous FBS proteins that may co‐aggregate during the EV isolation process. As an example, investigators identified acetylcholinesterase, a proposed marker for small EVs, as a likely non‐EV co‐aggregate derived from serum, rather than being associated with cell‐derived EVs (Liao et al., 2019). Taken together, direct usage of native FBS as a culture media supplement provides major consequences and potential for misinterpretations of EV analyses.

## 4 EV‐DEPLETED FBS: AN ALTERNATIVE AND ASSOCIATED PITFALLS

Thery et al. (Thery, 2006) proposed the use of either 1) serum‐free media; 2) 1% bovine serum albumin instead of whole FBS; or, 3) FBS EV‐‘depletion’ protocols, termed EV‐depleted FBS, if the cultured cells require serum supplementation for their growth. To be described throughout this manuscript, it is important to highlight that EV‐depleted FBS is not ‘EV‐free’ FBS media as these protocols never 100% deplete FBS‐derived EVs. Therefore, we will use the term EV‐depleted FBS for when any attempt to deplete FBS‐derived EVs has been performed. The gold standard for FBS EV‐depletion continues to include diluting FBS media and performing high‐speed ultracentrifugation (UC), removing the contained EVs within the pellet, and using the supernatant as the media supplement (Thery, 2006). Of note, performing UC on non‐diluted FBS is problematic, since the contained elevated levels of lipids, proteins, and lipoproteins tend to promote aggregation, leaving a less than optimal supernatant for use as a supplement (Thery, 2006). Additionally, during the UC depletion process, free or aggregated growth factors and other proteins may also be removed/reduced due to their similar density as EVs. This removal may also modulate the ability of the FBS to support cell growth (Lehrich et al., 2018). Therefore, it is important to consider this as a potential confounder in experiments comparing EV‐depleted FBS versus native FBS. Performing experiments that compare across multiple FBS EV‐depletion methods is vital as some methods deplete FBS‐derived EVs based on density, while others are based on size. Recently, commercial products are available that are putatively depleted of FBS‐derived EVs; however, the exact protocols are not specified (most utilizing polymer precipitants or ultrafiltration), and investigators should utilize these with caution.

Since these original FBS EV‐depletion protocols were proposed, other researchers have used EV‐depleted FBS media in their in vitro investigations. Unfortunately, a growing number of publications have highlighted differing cellular responses to reductions in presumed FBS‐derived EV levels in the culture medium through analytical evaluations between cultured cells in native and EV‐depleted FBS media (Table 1).

**TABLE 1:** Summary of main findings from FBS EV‐depleted protocols investigations

Study Author (Journal)	FBS EV‐depletion protocol	FBS EV‐depletion efficiency	Cell Type(s) subjected to depleted FBS	Impacts on cell growth and viability	Impacts on cell phenotype and behaviour	FBS EV RNA/exRNA contamination
Ochieng et al., 2009 (*Experimental Cell Research*)	FBS diluted 1:1; sequential centrifugation at 20,000 g (time undefined), 100,000 g (10‐h); pellet UC at 200,000 g (1‐hr) and dissolved in basal media	Not investigated	Breast carcinoma cell line (MDA‐MB‐435)	In FBS EV‐enriched media, cells formed 3D colonies; EV‐depleted media cells grew as a monolayer	FBS EVs facilitate anchorage independent growth of breast carcinoma cells and discharged reducing metabolites; FBS EVs taken up by breast carcinoma cells and traffic to the late‐endosome; EVs were recycled by cells; activate MAP kinases	Not investigated
Shelke et al., 2014 (*Journal of Extracellular Vesicles*)	10% FBS media UC at 120,000 g (18‐h)	EVs still detected by electron microscopy after 18‐h UC	Lung cancer epithelial cell line (A549)	Not investigated	Lack of cell migration in EV‐depleted FBS; addition of FBS EV pellet resulted in dose‐dependent increases in cell migration	18‐hr UC reduced RNA by 95%, while 1.5‐h UC reduced RNA by 50% with dilution not making a significant difference
Beninson et al., 2015 (*Immunology Letters*)	Exo‐FBS™ System Biosciences, Inc., SBI; Palo Alto, CA)	Nanoparticle concentration of exosome‐like (50‐100 nm) were reduced in Exo‐FBS™ using nanoparticle tracking analysis (Nanosight)	Primary rat peritoneal macrophages	Cell viability (cell death) measured through lactate dehydrogenase assay kit demonstrated similar results between FBS conditions	Reduced concentration of cytokines (IL‐1ß) following LPS challenge in non‐depleted FBS compared to Exo‐FBS™; this effect was mimicked with addition of isolated FBS EVs in Exo‐FBS™	Not investigated
Eitan et al., 2015 (*Journal of Extracellular Vesicles*)	FBS diluted 1:3; sequential centrifugation at 2000 g (10 min), 10,000 g (40 min), 120,000 g (1‐6 h); filtered with 0.22μm filter	Nanoparticle concentration (defined as size range of 40‐400 nm) reduced by 2.6‐fold by 1‐h of UC and 7‐fold by 6‐h	Human U87 glioblastoma, human embryonic kidney (HEK). 293t cells, HeLa cells, human SH‐SY5Y neuroblastoma cells, and mouse N2a neuroblastoma cells	All cell lines except U87 glioblastoma exhibited slower growth (measured via cell number) and cell viability (MTS assay); cell growth rescued with addition of isolated FBS EVs	FBS EVs labelled with PKH27 (red dye) were added to N2a, U87, and HEK cell lines labelled with PKH67 (green) and observed FBS EV uptake via endocytosis to the lysosome	Not investigated
Angelini et al., 2016 (*BioImpacts*)	1) FBS filtered with 0.22μm filter; centrifuged at 16,5000 rcf (18‐h)	Not investigated	Human cardiac progenitor cells	UC FBS and Exo‐FBS™ affected cell proliferation and can be rescued with addition of isolated FBS EVs in a dose‐dependent way	UC FBS and Exo‐FBS™ affected cell phenotype, including cell size (smaller diameter) but morphology not altered; UC FBS and Exo‐FBS™ affected cell genotype, including reduced extracellular matrix production and lower levels of Ki‐67 and THY‐1	Not investigated
	2) Exo‐FBS™ System Biosciences, Inc., SBI; Palo Alto, CA); filtered with 0.22μm filter					
Aswad et al., 2016 (*BMC Biotechnology*)	FBS diluted to 20%; UC at 100,000 g (overnight ∼18‐h); filtered with 0.22 μm filter; diluted to final 10% FBS	Not investigated	Mouse cell line C2C12, rat cell line L6, and human primary myoblasts	Myoblast proliferation reduced in EV‐depleted FBS	Cell proliferation and EV formation/ trafficking genes were downregulated in cells cultured in EV‐depleted media compared to non‐depleted media; myoblasts also expressed premature levels of genes important for cell differentiation	FBS EVs contain miRNAs participating in muscle myogenesis
Wei et al., 2016 (*Scientific Reports*)	FBS UC at 100,000 g (80 min, 5‐h, or 24‐h)	After 24‐h, 19%‐33% of RNA was removed from FBS; most of the RNA is presumed to be with non‐vesicular components of the FBS	U251 and 20/3 human glioma cells, GL261 mouse glioma cells, mouse embryonic fibroblast cells (MEF), CMT‐93 mouse rectum cells, RAW264.7 mouse macrophage cells, and EL4 mouse T lymphocytes	Not investigated	miR‐1246 (not expressed in mouse/rat) but highly expressed in bovine was detected in mouse cell lines cultured in FBS; cell lines processed bovine exRNAs (vesicular and non‐vesicular associated) which can affect genotype of the cultured cell	>70% of RNA species remain in EV‐depleted FBS; miR‐122, miR‐451a and miR‐1246 may be false annotations as cell enriched transcripts (and be FBS contamination); sequence homology between bovine and human/mouse transcriptome
Liao et al., 2017 (*Scientific Reports*)	1) FBS diluted 1:4 with basal medium at 110,000 g (18 h); filtered with 0.22μm filter	Greater significant depletion with Gibco serum and lowest variability in particle counts via nanoparticle tracking analysis; UC serum concentrations varied by lot number and UC run	H9 and PM1 (T‐lymphocytic lines); ACH‐2 (chronically HIV‐1‐infected T‐lymphocytic lines); U1 (promonocytic line); TZM‐bl and HEK‐293T cells; primary CD4+ T‐cells	Cell viability affected for H9 and PM1 when cultured in both EV‐depleted FBS conditions	Gibco EV‐depleted FBS correlated with increased HIV‐1 infectivity; HIV‐1 release, HIV‐1 production, increased cell aggregation and syncytium formation all significantly increased in infected cells; EV pellet rescue experiments lowered virus production levels; Cells cultured in EV‐depleted FBS had increased expression of surface/adhesion protein molecules, cellular basal and maximal respiration decreased, and ATP production decreased	No significant differences in miRNA expression patterns across cell types grown in EV‐depleted FBS conditions; gene ontology analysis demonstrated in EV‐depleted FBS conditions significant increases in lipid and sterol synthesis pathways
	2) Gibco Exosome‐Depleted FBS (Thermo Fisher, USA)					
Kornilov et al., 2018 (*Journal of Extracellular Vesicles*)	1) non‐diluted FBS UC at 26,000 rpm (121,896 g) (19‐h); filtered with 0.22 μm filter	Particle concentrations similar between ultrafiltered FBS and Exo‐FBS™ with UC lower than non‐depleted FBS; HSP70 absent and CD71 faint band via western blot; no EV RNA peaks for ultrafiltered FBS while small traces of EV RNA for UC FBS and Exo‐FBS™ (miRNEasy Serum/Plasma. kit [Qiagen])	Human adipose tissue mesenchymal stem cells; human prostate cells; human renal cells; mouse 3T3 cells; Osteosarcoma cell line HOS143b; prostate cancer cell line PC‐3; Oral cancer cell line HSC3	All EV‐depleted media conditions displayed normal levels mesenchymal stem cell proliferation compared to non‐depleted FBS; cell proliferation was physiologically acceptable in cancer cell lines with ultrafiltration protocol; no significant difference in reactive oxygen species release between ultrafiltered and non‐depleted FBS cell cultures	All EV‐depleted media conditions displayed normal mesenchymal stem cell morphology (i.e., cell body, size, shape, and processes) compared to non‐depleted FBS; osteogenic differentiation was not affected in the different EV‐depleted media conditions; cell‐derived EV release was not affected by the EV‐depleted media conditions	All EV‐depleted media conditions contained some residual RNA present (soluble RNA in ultrafiltered FBS)
	2) non‐diluted FBS ultrafiltered at 3000 g (55 min) with Amicon ultra‐15 centrifugal filter					
	3) Exo‐FBS™ System Biosciences, Inc., SBI; Palo Alto, CA)					
Lehrich et al., 2018 (*International Journal of Molecular Sciences*)	1) FBS diluted to 20%; UC at 100,000 g (18‐h); filtered with 0.22 μm filter; diluted to final 10% FBS	18‐h UC allowed removal of non‐exosomal (> 150 nm) EVs, while smaller (75–250 nm) exosome‐like particles remain (70% EV reduction); Exo‐FBS™ leaves a heterogeneous mixture of residual EVs (75–500 nm) (75% EV reduction) as confirmed with nanoparticle tracking analysis (ZetaVIEW)	Primary rat astrocytes	70% cell confluence for 18‐h UC and 50% cell confluence for Exo‐FBS™ cultured cells; Both EV‐depleted FBS conditions provided poor cell viability, but 18‐h UC of FBS provided significantly better cell viability compared to Exo‐FBS™	Morphological differences included astrocyte cell body size decreases and floating/dead astrocyte cell increases	Not investigated
	2) Exo‐FBS™ System Biosciences, Inc., SBI; Palo Alto, CA)					
Driedonks et al., 2019 (*Journal of Extracellular Vesicles*)	FBS was either a) diluted to 30%, or b) diluted to 10%; UC at 100,000 g (15‐18‐h); prepared by either decanting or pipetting	EV‐depletion reduced EV counts by 90%; FBS RNA was reduced more (and pelleted better) in more dilute FBS; decant method left more RNA (Y‐RNA and 7SL) in the supernatant used for culture	Human Embryonic Kidney (HEK293T) cells and murine B‐lymphoblast (A20) cells	Not investigated	Different RNA species displayed different fold reductions depending on the distinct EV‐depletion protocol and not affected by transcript quantity before EV‐depletion	RNAs present in the EV‐depleted media may contaminate the EV transcriptome produced by the cultured cells through uptake
Mannerstrom et al., 2019 (*Scientific Reports*)	1) non‐diluted FBS UC at 26,000 rpm (121,896 g) (19‐h); filtered with 0.22μm filter	Transmission electron microscopy showed no vesicles present in ultrafiltered FBS, but present in normal FBS, UC FBS, and Exo‐FBS™; anti‐transferrin receptor/CD71 detected via western blot in normal FBS and Exo‐FBS™, but not UC FBS or ultrafiltered FBS; nanoparticle tracking confirmed the western blot and showed very little particles in ultrafiltered FBS	Not used	Not investigated	Not investigated	Small non‐coding RNA (ncRNA) sequencing data demonstrated that there were some degree of RNA species remaining regardless of the EV‐depletion protocol; the ultrafiltration approach depleted the most bovine miRNA species; RNA species arduous to remove included ‐ miR‐122, miR‐203a, nRNA, Y RNA, snoRNA, and piRNA; no media RNA free
	2) non‐diluted FBS ultrafiltered at 3000 g (55 min) with Amicon ultra‐15 centrifugal filter					
	3) Exo‐FBS™ System Biosciences, Inc., SBI; Palo Alto, CA)					

## 5 FBS EV‐DEPLETION PROTOCOL EFFICIENCIES

**FIGURE 2:**
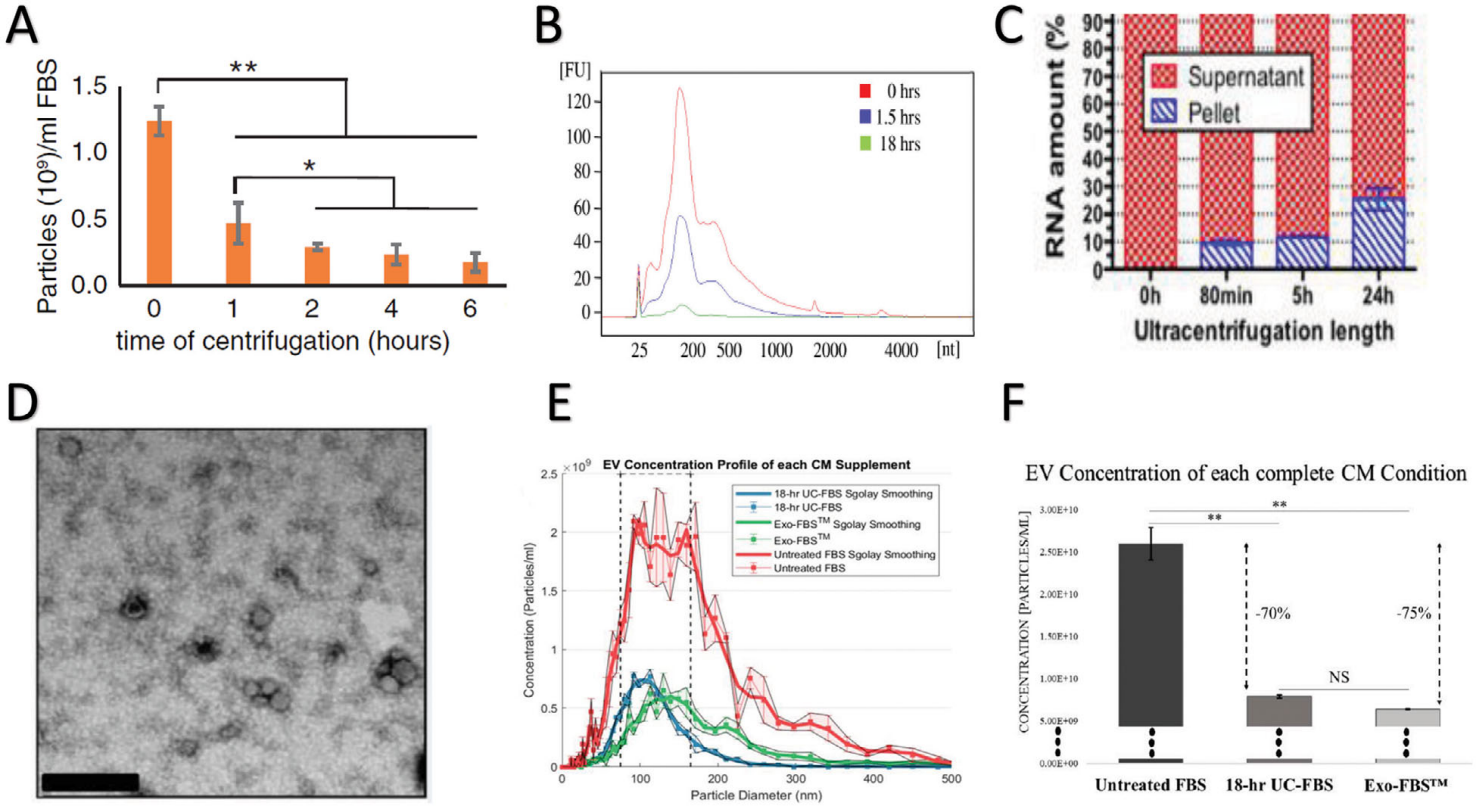
FBS EV‐depletion Protocol Efficiencies. (A) FBS‐derived EV particle depletion performed at different time points quantified using NTA with a NanoSight NS‐500 instrument (n = 3 replicates). UC spin occurred at 120,000 x g following a 10,000 x g initial spin for 40 min. Reproduced from Eitan et al. J Extracell Vesicles, 2015. 4: p. 26373. (B) FBS exRNA remaining following depletion over time. Reproduced from Shelke et al. J Extracell Vesicles, 2014. 3. (C) FBS exRNA remaining in supernatant following UC depletion over time. Reproduced from Wei et al. Sci Rep, 2016. 6: p. 31175. (D) Presence of small EVs was determined by transmission electron microscopy. Vesicles are from the pellet preparation of EV‐depleted FBS following an 18‐h UC. Scale bar is 200 nm. Reproduced from Shelke et al. J Extracell Vesicles, 2014. 3. (E) Native FBS, UC EV‐depleted FBS media, and Exo‐FBS™ particle size distributions. Reproduced from Lehrich et al. Int J Mol Sci, 2018. 19(11). (F) Native FBS, UC EV‐depleted FBS media, and Exo‐FBS™ particle concentration differences. Reproduced from Lehrich et al. Int J Mol Sci, 2018. 19(11)

Many studies have assessed EV depletion efficiency through reductions in either particle numbers or putative EV‐associated RNAs. Size‐ and concentration‐based estimations typically include nanoparticle tracking analysis (NTA) or tunable resistive pulse sensing (TRPS). However, these techniques lack specificity and sensitivity, and are not able to distinguish between EVs and other EV‐like nanoparticles (e.g., lipoprotein particles) (Karimi et al., 2018), as NTA may detect concentrations of contaminant low‐density lipoproteins (Gardiner et al., 2013). Nanoparticle depletion efficiency is affected by a variety of factors, including UC speed, time, serum dilution, and/or usage of polymer precipitants. Increasing the UC (@120,000 g diluted 1:3) time (e.g., from 2‐ to 6‐h) is known to provide greater nanoparticle depletion (i.e., 7‐fold reduction) in the size range of 50–500 nm as evidenced by NTA using a NanoSight NS‐500 instrument (Figure 2 A) (Eitan et al., 2015). Additionally, other investigators demonstrated that an 18‐h UC (@120,000 g diluted 3:7) removes up to 95% of FBS RNA species compared to only 50% with a 1.5‐h UC spin (Figure 2 B, C, D) (Shelke et al., 2014; Wei et al., 2016). In this study, the FBS EV pellet (isolated from EV‐depleted FBS) was treated with proteinase K and RNase to exclude other particle‐associated RNAs based on the assumption the EV‐RNAs are protected within the vesicle. However, the amount of residual EVs in the EV‐depleted supernatant was not measured, which makes it difficult to draw definitive conclusions on EV‐depletion efficiency (Shelke et al., 2014). Some reports suggest that polymer precipitant methods provide the greatest EV‐depletion and reduced variability, while UC methods provide high variability based on each run, batch, and lot differences, and thereby affect final nanoparticle concentrations (Liao et al., 2017). Similarly, quantitative results from our group reported that an 18‐h UC (@100,000 g diluted 1:5) resulted in removal of larger (> 250 nm) nanoparticles, while smaller (75–250 nm) nanoparticles remained as measured via NTA with a ZetaVIEW instrument size ranging limits from 50–500 nm (Figure 2 E). Moreover, polymer precipitants, in our hands, resulted in a more heterogeneous mixture of residual nanoparticles (75–500 nm) in the media supplement. Despite both FBS EV‐depletion methodologies producing 70% and 75% reductions in nanoparticles, for UC and commercial precipitants, respectively, quantitative analyses indicate significant remaining quantities (10^9^ particles/ml) of nanoparticles within the EV‐depleted FBS media conditions (Figure 2 F) (Lehrich et al., 2018).

Depending on the exact depletion protocol, various nanoparticles, possibly also including EVs, remain abundant in the EV‐depleted FBS media. EVs (1.10‐1.19 g/ml) can be separated based on density compared to chylomicrons, very low density lipoprotein (VLDL), and low density lipoprotein (LDL) particles (< 1.063 g/ml), however overlap in density with high density lipoproteins (HDL) (1.063‐1.21 g/ml), making their separation from EVs size‐dependent (HDL: 4–10 nm) (Brennan et al., 2020). Therefore, since both EV and lipoproteins may be detected by nanoparticle size‐based analyses, and both are carriers of exRNAs (Vickers et al., 2011), particle counts and total RNA quantification cannot specifically address EV‐depletion from FBS. Instead, FBS EV‐depletion efficiency should be determined by quantifying EV‐specific protein markers (e.g., CD9, CD63, CD81) via Western Blot (or proteomic assays) in parallel with unconditioned medium controls, including non‐depleted FBS, EV‐depleted supernatant, and FBS‐EV pellet samples. Additionally, amounts of non‐EV nanoparticles that overlap in size and density may be determined by quantifying lipoprotein markers (e.g., ApoA‐1, ApoB100, ApoB‐48, ApoE) in these samples (Brennan et al., 2020; Zhang et al., 2020). Overall, sequential combinations of EV isolation techniques (based on size, density, zeta potential (Zhang et al., 2020), or antibody binding (Mørk et al., 2017)) allow the isolation of nanoparticle populations of interest.

## 6 EXTRACELLULAR RNA EXISTENCE WITHIN EV‐DEPLETED FBS

**FIGURE 3:**
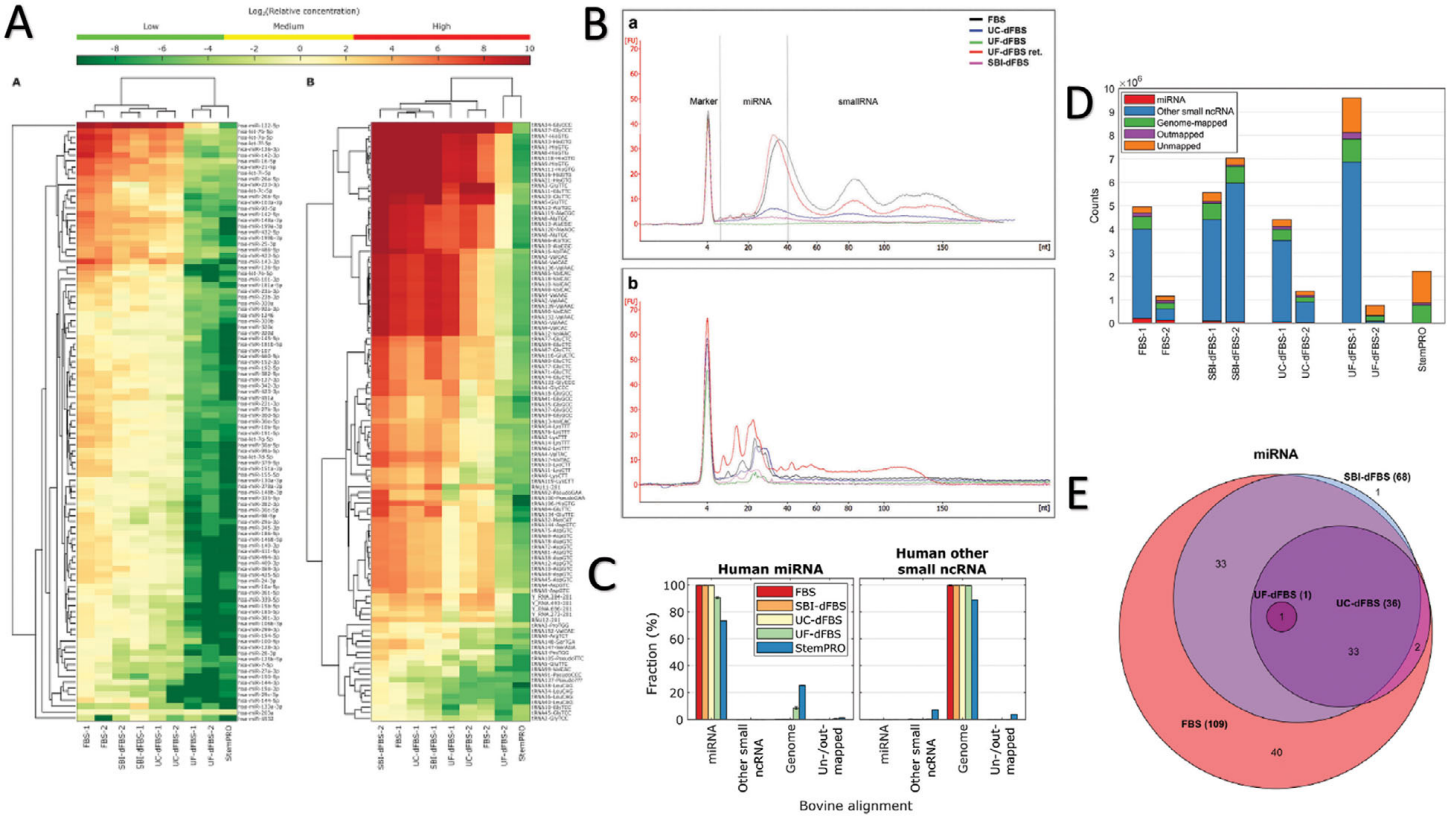
Extracellular RNA Existence within EV‐depleted FBS Media. (A) Heatmap demonstrating miRNAs and small noncoding RNAs that were abundant in different FBS media conditions (native, Exo‐FBS™, ultracentrifugation, and ultrafiltration). Reproduced from Mannerström et al. Sci Rep, 2019. 9(1): p. 5538. (B) Top; RNA profiles in different FBS conditions from the EVs isolated from the retentate. Bottom; RNA profiles in the FBS media preparations utilized. Reproduced from Kornilov et al. J Extracell Vesicles, 2018. 7(1): p. 1422674. FBS transcript sequence counts mapped to (C) bovine and (D) human reference genomes for varying RNA species in each sample (native, Exo‐FBS™, ultracentrifugation, and ultrafiltration). Reproduced from Mannerström et al. Sci Rep, 2019. 9(1): p. 5538. (E) The Euler diagram provides visual representation of the total quantity of distinct RNAs per media condition. Reproduced from Mannerström et al. Sci Rep, 2019. 9(1): p. 5538

For investigations of in vitro‐derived EVs, exRNA introduced from FBS should be seriously considered (Figure 3 A). Serum contains a variety of carriers of exRNA including EVs, lipoproteins, and ribonucleoprotein complexes (RNPs) (Tosar et al., 2018) (Figure 3 B). FBS EV‐depletion protocols, namely UC, are primarily designed to remove EVs and EV‐like particles, leaving uncertainty as to the extent of remaining exRNA carriers present in the media supplement. Such remaining RNA complexes may confound a variety of experimental results, but especially those assessing EV‐associated RNA species (Figure 3 C, D) (Wei et al., 2016). One study with RNA‐sequencing of EV‐depleted FBS media reported that even after a 24‐h UC (@100,000 g undiluted), a major proportion of FBS‐derived exRNA species remain in solution (Wei et al., 2016). Though contrary to the prior study (Shelke et al., 2014), this may be due to differing spin speeds, dilution factors, and/or RNA quantification techniques. Moreover, this study found that miR‐122, miR‐451a, which are conserved between humans and cows, are highly abundant in native FBS and remain in the supernatant after EV‐depletion protocols (Wei et al., 2016). However, it is not completely understood which RNA types are associated with EVs or with other exRNA carriers, and which exRNA carriers remain in solution after FBS EV‐depletion.

In fact, it remains difficult to separate individual subclasses of exRNA carriers from plasma or serum (Srinivasan et al., 2019). Argonaute2 complexes are a major reservoir for miRNAs in plasma or serum (Arroyo et al., 2011), and are known to be incompletely removed via UC. Therefore, this class of exRNAs may not be efficiently removed from EV‐depleted FBS (Turchinovich et al., 2011), but may be co‐isolated with in vitro‐derived EVs during polymer‐based EV isolation. HDL has been confirmed as a carrier for miRNA, lncRNA, tRNA or rRNA (Allen et al., 2018), and due to their similar density as EVs, exRNAs carried on HDLs may co‐precipitate following UC (e.g., density gradient or sucrose cushion). However, the degree of depletion of exRNA carriers achieved in EV‐depleted FBS is rarely quantified. It is likely that varying but substantial quantities of exRNA species/carriers (EV‐associated or non‐EV‐associated) remain following EV‐depletion protocols. Careful design of EV isolation methods may improve the purity of in vitro‐derived EVs and exclude a majority of FBS‐derived exRNA carriers (Figure 3 A, E) (Karimi et al., 2018; Mannerström et al., 2019; Onódi et al., 2018). Inclusion of parallel processing controls of non‐conditioned FBS‐supplemented culture media to compare with the cell‐derived EV fraction may be another solution to assess RNA background levels from potential contaminant exRNAs introduced by EV‐depleted FBS (Auber et al., 2019; Driedonks et al., 2019). Further, batch‐to‐batch variations of FBS should be considered when vendors or lots are switched in a laboratory. For additional considerations regarding FBS‐derived exRNA contamination and other sources of common laboratory RNA contamination, we refer the reader to the following articles (Das et al., 2019; Murillo et al., 2019; Srinivasan et al., 2019; Tosar et al., 2017).

## 7 IMPAIRED CELL GROWTH IN FBS EV‐DEPLETED MEDIA

Many experiments suggest that FBS‐derived EVs (or EV‐like particles) in culture media contribute yet undefined factors important for cultured cell growth and viability. One of the first reports demonstrated that the FBS‐derived EV pellet facilitated anchorage‐independent growth of breast carcinoma cells (Ochieng et al., 2009). Another group tested a variety of different cell lines (i.e., U87 glioblastoma, HEK‐293T, HeLa, SY5Y human neuroblastoma, and N2a mouse neuroblastoma cells) grown in native and EV‐depleted FBS media and observed that growth rates and cell viability were substantially reduced in the EV‐depleted FBS media for all the cell lines tested, except the U87 cell line. Remarkably, if the FBS‐derived EV pellet was ‘spiked‐in’ to the culture media, there is an apparent salvage of growth (Eitan et al., 2015).

**FIGURE 4:**
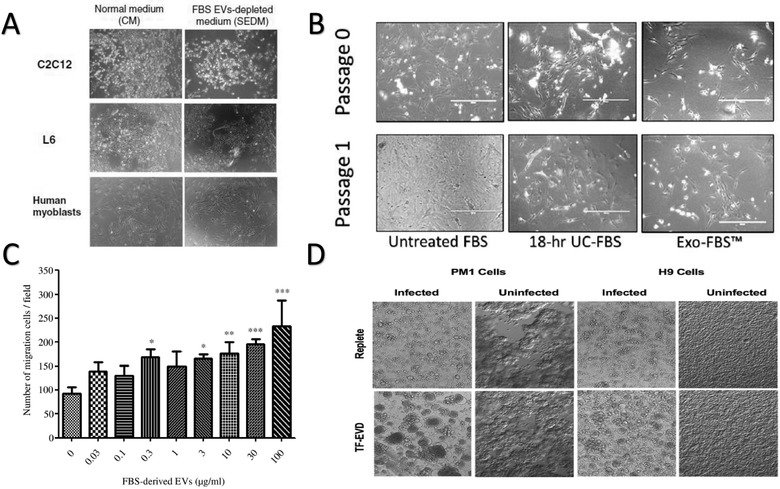
Deterred Cell Growth in EV‐depleted FBS Media. (A) Human primary myoblasts cultured in native or EV‐depleted FBS media. Reproduced from Aswad et al. BMC Biotechnol, 2016. 16: p. 32. (B) Mouse primary astrocytes cultured in native or EV‐depleted FBS media (via ultracentrifugation or Exo‐FBS™). Reproduced from Lehrich et al. Int J Mol Sci, 2018. 19(11). (C) Illustration that addition of FBS‐derived EVs to EV‐depleted FBS media stimulates migratory cell phenotype in an epithelial cell line. Reproduced from Shelke et al. J Extracell Vesicles, 2014. (D) Cellular phenotypic changes in HIV‐1 infected H9 and PM1 cell cultures under different FBS media conditions (replete = native; TF‐EVD = EV‐depleted FBS media). Adapted from Liao et al. Sci Rep, 2017. 7(1): p. 2558

These negative cell physiological effects associated with EV‐depleted FBS media have also been illustrated in primary cell culture systems, including primary human myoblasts (Figure 4 A) (Aswad et al., 2016), primary mouse astrocytes (Figure 4 B) (Lehrich et al., 2018), and cardiac progenitor cells (Angelini et al., 2016). The latter investigation demonstrated that in human cardiosphere‐forming cells, FBS‐derived EVs appear to modulate cell proliferation, migration, and differentiation. Additionally, cardiosphere structure is affected with differences in sphere volume, overall production, and extracellular matrix generation (Angelini et al., 2016). Lastly, our group revealed that primary mouse astrocytes cultured in EV‐depleted FBS media demonstrate suboptimal growth and viability compared to culture in native FBS media (Figure 4 B) (Lehrich et al., 2018).

Based on the literature and our own experiences, therefore, the impaired cell growth and viability observed in EV‐depleted FBS is likely due to removal of FBS‐derived EVs and/or other co‐isolated particles. In a series of experiments studying myoblast proliferation, researchers demonstrated that genes important for cell proliferation (i.e., CCND1, SIRT1) were downregulated in EV‐depleted FBS media (Aswad et al., 2016). Additionally, FBS‐derived EV cargo molecules such as Wnt, TGFß, HSP, sonic hedgehog, SOD, Catalase and survivin may also contribute to these observed cell growth differences (Auber et al., 2019; Eitan et al., 2015). Therefore, researchers are encouraged to properly control for the cell biological influences and their effects on downstream analyses. We suggest that cell proliferation and viability assays be utilized to monitor the effects of cell growth/death, along with the use of an EV potency assay for examining the EVs produced under these ‘stressed’ physiological conditions providing a preclinical assessment of their therapeutic efficacy, dosing, and biological function (Bobis‐Wozowicz et al., 2017; Willis et al., 2017).

## 8 OTHER PHENOTYPIC DIFFERENCES IN FBS EV‐DEPLETED MEDIA

In addition to impaired cell growth, other investigations have observed induction of specific cellular phenotypes (i.e., alterations in migration, differentiation, inflammation, and secretion) when cultured in EV‐depleted FBS media. An airway epithelial model demonstrated that compared to native FBS, EV‐depleted FBS media restrained cell migration, which could be salvaged through the direct addition of the isolated FBS‐derived EV pellet in a dose‐dependent manner (Figure 4 C) (Shelke et al., 2014). A cell differentiation study, utilizing primary cultures of human myoblasts (Aswad et al., 2016), demonstrated that specific genes were differentially expressed when cultured in EV‐depleted FBS media. Remarkably, these investigators observed that switching from EV‐depleted to native FBS media reversed the induced phenotypic characteristics, thereby reinforcing the role that FBS EV‐depletion protocols modulate cultured cell behaviour (Aswad et al., 2016).

The impact of FBS EV‐depletion protocols on immune cell function and subsequent inflammatory response has also been characterized (Beninson & Fleshner, 2015). Specifically, primary macrophages cultured in EV‐depleted FBS media significantly increase release of pro‐inflammatory cytokines when stimulated with lipopolysaccharide (Beninson & Fleshner, 2015). Similarly, another report observed that HIV‐infected T‐lymphocytic cell lines cultured in EV‐depleted FBS media showed increased HIV infectivity, production, release, and cell aggregation and syncytium formation (Figure 4 D) (Liao et al., 2017). Also, when cultured in EV‐depleted FBS media, the T‐cells had increased markers for surface/adhesion proteins, lower basal and maximal respiration, and lower ATP production (Liao et al., 2017). Given the widely reported evidence that HIV may hijack EV production and secretion pathways, these results highlight the possibility that conditions present in the EV‐depleted FBS media may modify the characteristics of EV production within certain cultured cells, and thereby affect downstream analyses. In support of these findings, genes associated with EV formation and trafficking (i.e., VPS37B, VPS4A) were distinctly downregulated when cells were cultured in EV‐depleted FBS media (Aswad et al., 2016). In summary, these findings lead us to strongly consider that, in response to the various depletion processes, EV‐depleted FBS media may influence certain parent cell phenotypes and possibly their qualitative and quantitative production of EVs, requiring careful consideration.

## 9 SERUM‐FREE MEDIA: INSTANT SWITCH AND CELLULAR STRESS

Specific in vitro EV researchers have utilized serum‐free media for EV isolation. However, multiple studies have demonstrated potential pitfalls when using serum‐free media (Gudbergsson et al., 2016; Potier et al., 2007; Sun et al., 2014; Zhu et al., 2006). Once cells reach the recommended 70%–80% confluence for EV isolation, aggressively switching from native FBS to serum‐free media may cause unintended cellular stress and autophagic flux (Wang et al., 2019), changes in the cellular phenotype, and potential alterations in EV cargo packaging and release mechanisms (Vallabhaneni et al., 2015). Additionally, there may be carryover of FBS‐derived EV and non‐EV components that persist despite the switch to serum‐free media (Auber et al., 2019; Mannerström et al., 2019).

Specifically, serum deprivation may induce cell death (in mesenchymal stem cells) (Potier et al., 2007; Zhu et al., 2006), or alter the concentrations, yield, and protein topography of in vitro‐derived EVs (Gudbergsson et al., 2016; Sun et al., 2014). When serum concentrations are reduced from 10% to 1%, the size distribution, total quantity, and protein composition of in vitro‐derived EVs were different (Sun et al., 2014). Additionally, EV secretion is partially facilitated through the autophagy‐lysosomal pathway (Buratta et al., 2020; Wang et al., 2019; Xu et al., 2018), where specific RNA‐binding proteins are expressed during serum starvation (Leidal et al., 2020). Additionally, cellular stresses introduced by serum‐free media may induce specific intracellular signalling cascades associated with EV biogenesis (i.e., G‐protein and GTPase/Ras‐related) (Li et al., 2015). Moreover, cellular stresses introduced by different serum concentrations may activate the NF‐κB pathway and contribute to EV‐dependent oncogenesis (Sun et al., 2014). In fact, one study observed in vitro‐derived EVs from serum‐deprived mesenchymal stem cells carried tumour‐supportive miRNAs and proteins, that supported growth of xenografted breast tumours (Vallabhaneni et al., 2015). Conversely, one study observed little alteration in cell glycosylation and viability under serum depletion in a human gastric cancer cell line (Freitas et al., 2019), yet another suggested that cellular stresses may not affect EV size and concentration in human microvascular endothelial cells (De Jong et al., 2012). Nonetheless, cell physiological changes and EV release profiles seem need to be considered for cells cultured in serum‐free media and tend to differ based on cell types (immortalized versus primary cells).

In addition, serum‐free media may not be completely devoid of contaminants (Auber et al., 2019). Although serum‐free media will not contain FBS‐derived EVs, there may still exist EV‐like particles detected via nanoparticle tracking analysis (e.g., protein aggregates), particularly in serum‐replacement supplements compared to non‐supplemented medium, that may interfere with downstream EV analyses (Lee et al., 2019). The UC pellet from serum‐free media has been shown to contain protein aggregates and vesicular structures when visualized under transmission electron microscopy, along with displaying the presence of transferrin receptor/CD71 on western blot (Mannerström et al., 2019). Moreover, these defined serum‐free media conditions contained detectable RNA species, which may be derived from other source materials (e.g., amino acids, vitamins) (Mannerström et al., 2019). Altogether, these results suggest that while FBS‐derived EVs are absent in serum‐free media, preventing RNA/protein contamination from other sources remain a potential concern.

## 10 POTENTIAL SOLUTIONS TO REFINING CULTURE PROTOCOLS

The EV community (Consortium, 2017; Lötvall et al., 2014; Théry et al., 2018) and our group (Lehrich et al., 2019) has advocated for additional transparency in the reporting of FBS EV‐depletion protocols, striving towards improved reproducibility. Our group has encouraged similar efforts in the field of blood‐based metabolomic biomarkers (Fiandaca, 2018; Gross et al., 2018). A recent study suggests that current in vitro EV isolation protocols may be readily translatable to clinical use, based on available methods (Pachler et al., 2017), with another study providing a standardized operating procedure (SOP) for manufacturing clinical‐grade EV therapeutics (Mendt et al., 2018). In this application, cells are often genetically modified for overexpression of specific EV targets, and are consistently maintained in a defined condition (e.g., suspension culture, serum‐free, or chemically‐defined media) for upscaling EV production (Gimona et al., 2017). Therefore, cell physiological changes may not be a primary concern provided the profile of manufactured therapeutic EVs is well assessed for toxicity and batch‐to‐batch consistency. However, these methods are typically employed for cell line cultures (many do require serum for growth as well), and may be limited in the case of primary cell cultures (Lener et al., 2015). A comparison of utilizing EV‐depleted FBS or serum‐free media is briefly summarized in Table 2.

**TABLE 2:** Comparison of utilizing EV‐depleted FBS and serum‐free media conditions in terms of therapeutic applications

		EV‐depleted FBS media	Serum‐free media
Culture preparation		Various depletion protocols and time consuming, or commercial options	Defined and simple
Exogenous EVs contamination		Probably yes	None
exRNAs contamination		Yes (Wei et al., 2016; Turchinovich et al., 2011)	No for basal medium, but Yes for chemically‐supplemented medium (Auber et al., 2019)
Cell physiology	Cell lines	Affected (Eitan et al., 2015)	Affected, but may be adapted (Lee et al., 2019; Li et al., 2015)
	Stem/primary cells	Affected (Liao et al., 2017; Driedonks et al., 2019; Tosar et al., 2017)	Affected, may need addition of growth factors (Vallabhaneni et al., 2015; Zhu et al., 2006)
EV release		May be affected (Wei et al., 2016; Driedonks et al., 2019)	Affected (Gudbergsson et al., 2016; Li et al., 2015; Sun et al., 2014), yet may be cell‐type dependent (De Jong et al., 2012)
Cell‐derived EV Profile		Need to determine	Affected (Gudbergsson et al., 2016; Sun et al., 2014), yet may be adapted with consistency and without undermining therapeutics (Gimona et al., 2017)
Cost		Cost usually high when preparing or purchasing EV‐depleted FBS	Less when using basal medium, and may increase when using chemically‐supplemented medium

One group has suggested that ultrafiltration (i.e., Amicon ultra‐15 centrifugal filters), rather than UC or polymer precipitant methods, is a more efficient FBS EV‐depletion methodology, and provides an environment for proper maintenance of cell growth and viability (Kornilov et al., 2018). Additionally, another group suggested that technical modifications to existing FBS EV‐depletion protocols, such as supernatant removal techniques (e.g., decant versus pipette), or secondary density gradient UC to potentially separate cell‐derived EVs from non‐EV contaminants, can substantially affect the efficiency of those methods (Driedonks et al., 2019). These methods, however, have not yet been widely adopted, verified, and standardized, along with potential reporting errors in depletion efficiency due to technique limitations in accurately detecting nanoparticles (Akers et al., 2016; Maas et al., 2015; Van Der Pol et al., 2014; Vestad et al., 2017). Therefore, there remains a need to develop serum‐ and xeno‐free, customizable, chemically‐defined media for various cell types to allow more rigorous in vitro EV investigations. Prior research has observed that xeno‐free substances, such as human sera or platelet lysate, can be used as a source of nutrients for cultured cells, analogous to FBS. Both additives, however, provide their own exogenous EVs (Laner‐Plamberger et al., 2015; Pachler et al., 2017; Witwer et al., 2019), with the ISEV consortium recommending the use of culture media conditions devoid of platelet lysate, bile salts, and pituitary extract, to prevent this contamination (Théry et al., 2018). However, in cases where this is not feasible, a strict culture ‘history’ is recommended with the use of proper unconditioned medium controls to assess the amount of exogenous contaminants that are co‐purified with EVs of interest. Our group has favoured using serum‐free culture media, supplemented with defined substrates necessary for growth, when analyzing in vitro‐derived EVs (Lehrich et al., 2018; Lehrich et al., 2019). Specifically, there exist databases (https://fcs‐free.org/) to aid in defining available serum‐free culture media alternatives (Brunner, 2010; Gstraunthaler, 2003).

Currently there are a plethora of serum supplementation issues to be resolved for reproducible in vitro EV investigations. In addition to supporting efforts by the ISEV, we suggest two additional steps for reporting in vitro‐derived EV data. With the recent publication of the common repository of FBS proteins, we advocate for investigators to confirm that the putative proteins identified from isolated in vitro‐derived EVs be cross‐referenced with this database, and whenever possible, to provide additional quantitative measures of relative abundance (Shin et al., 2019). Widespread participation will ensure a more accurate interrogation of the cell‐derived EV proteome. Additionally, we support the suggestions set forth by Auber and colleagues (Auber et al., 2019), advocating for the reporting of deep sequencing and RNA‐seq data (both coding and noncoding RNA species) from unconditioned media controls, as a background reference for analyzing in vitro‐derived EVs, and for performing RNA‐seq to identify non‐vesicular exRNAs (Tosar & Cayota, 2018). Within this, genome sequencing for FBS components may be needed depending on the experimental application. The different techniques and protocols for EV proteomic profiling and exRNA isolation and extraction methods have been excellently detailed elsewhere (Bakr, 2018; Sinha, 2018). Moreover, normalization factors and complete process controls (i.e., unconditioned medium controls as a background reference) (Driedonks et al., 2019; Tosar et al., 2017) need to be developed for comparing across differing media conditions. Medium controls are especially important in the case where some isolation methods (along with technical expertise) may be more efficient at removing certain nanoparticle populations compared to others. Lastly, we encourage the field to refer to techniques from investigators within the therapeutic viral vector field, where more experienced strategies have been employed to avoid influence of FBS usage in the production, purification, and safety of therapeutic viral vectors, which may be adopted towards in‐vitro EV analyses. For instance, lenti‐ and retro‐viruses are purified from conditioned serum‐free medium (Cribbs et al., 2013), where viral release is attested and impact from cell starvation and stress is generally not observed. This is in line with the earlier discussion that for EV therapeutic intent, serum‐free culture may be employed given that the quality of EVs is established. Analogously, adeno‐associated virus (AAV) and adenovirus (AdV) are generated intracellularly within native FBS culture medium (Kimura et al., 2019). Regularly, to eliminate FBS‐derived contamination multiple steps/rounds of purification are involved, including density gradient UC, affinity chromatography, or size‐exclusion chromatography to ensure the removal of any non‐viral components or empty vectors (Kutner et al., 2009; Merten et al., 2014). We believe current best practices should include: 1) using sequential EV isolation protocols based on size and density (i.e., UC/UF/size‐exclusion chromatography); 2) extensive characterization of the final EV pellet in terms of size, morphology, RNA, and protein markers to ensure purity of EVs (i.e., tetraspanins) and removal of non‐EV‐contaminants (i.e., ApoA, ApoB, ApoE); and, 3) inclusion of unconditioned media controls as background reference standards. Establishing such purification standards would benefit the EV field where varying serum‐based culture protocols are still widely employed.

## 11 CONCLUSIONS

Currently, FBS as a culture media supplement contributes far too many elements, as discussed, for studying in vitro‐derived EVs. Importantly, current FBS EV‐depletion protocols lack the ability to significantly reduce the quantities of FBS‐derived EVs, exRNA species, protein‐RNA complex aggregates, and lipoproteins within EV‐depleted FBS media, which may contaminate downstream cell‐derived EV isolation. Additionally, such media contributes analytic elements with high variability and inconsistency, making comparable analyses difficult, if not impossible. Based on the literature, the EV field may benefit from the use of chemically defined, serum‐free, and xeno‐free media that not only is optimized for cell growth and viability for a variety of cell types, but also is free of exogenous contaminating FBS‐derived EVs and extracellular protein/exRNA species. Although achieving such a media standard is not in the near future, it will ultimately ensure proper isolation of in vitro‐derived EV populations that will lead to translatable clinical applications.

## CONFLICTS OF INTEREST

The authors report no conflict of interest concerning the materials or methods used in this study or the findings specified in this paper.

## SOURCE OF FUNDING

Research reported in this publication was supported by the National Institute of General Medical Sciences of the National Institutes of Health under Award Number T32GM008208 to Brandon M. Lehrich. The content is solely the responsibility of the authors and does not necessarily represent the official views of the National Institutes of Health.

## FINANCIAL INTERESTS

Patent applications are pending related to blood exosomal cargos as biomarkers of neurological disease by MSF and other co‐inventors.
